# Short-term maximal performance, alertness, dietary intake, sleep pattern and mood states of physically active young men before, during and after Ramadan observance

**DOI:** 10.1371/journal.pone.0217851

**Published:** 2019-06-04

**Authors:** Hsen Hsouna, Raouf Abdessalem, Omar Boukhris, Khaled Trabelsi, Lassaad Chtourou, Nabil Tahri, Florian A. Engel, Roy J. Shephard, Hamdi Chtourou

**Affiliations:** 1 UR15JS01: Education, Motricité, Sport et Santé (EM2S), High Institute of Sport and Physical Education, University of Sfax, Sfax, Tunisia; 2 Department of Gastroenterology and Hepatology, Hedi Chaker Hospital, Sfax, Tunisia; 3 Institute of Sport and Sport Science, Department Movement and Training, Heidelberg University, Heidelberg, Germany; 4 Faculty of Kinesiology and Physical Education, University of Toronto, Toronto, ON, Canada; 5 Institut Supérieur du Sport et de l’Education Physique de Sfax, Université de Sfax, Sfax, Tunisia; 6 Activité Physique, Sport et Santé, UR18JS01, Observatoire National du Sport, Tunis, Tunisia; Universidade Federal de Juiz de Fora, BRAZIL

## Abstract

**Purpose:**

To assess changes in short-term maximal performance, alertness, dietary intake, sleep pattern and mood states of physically active young men before (BR), during and after Ramadan observance.

**Methods:**

Twelve physically-active men (age: 21.9±2.4yrs, height:1.77±0.09m, body-mass: 72.6±7.8kg, exercising: ≥3h/week) performed the 5-jump and the digit-cancellation (alertness) tests 15-days BR, on the first (FR) and last 10-days of Ramadan (ER) and 10-days (AR10) and 20-days (AR20) after Ramadan. During each period, sleep pattern (Pittsburgh-Sleep-Quality-Index (PSQI)), mood states (Profile-of-Mood-States **(**POMS)) and dietary intake were recorded.

**Results:**

No significant changes in the 5-jump, digit-cancellation test and POMS parameters appeared during and after Ramadan relative to BR. However, the PSQI total score was lower during FR compared to AR10 (p<0.001). Specifically, the subjective sleep quality was lower (*i*) at BR compared to FR (p<0.05), AR10 (p<0.01) and AR20 (p<0.01) and (*ii*) at ER and AR20 compared to FR (p<0.05). The sleep duration (*i*) increased at FR (p<0.05) and (ii) decreased at AR10 (p<0.01) and AR20 (p<0.05) compared to BR. Sleep disturbances were significantly greater (*i*) at BR compared to FR (p<0.01), ER (p<0.01), AR10 (p<0.05) and AR20 (p<0.05) and (ii) at AR10 and AR20 compared to FR and ER (p<0.05). In terms of diet, the fractional contribution of carbohydrate (%) was lower and the dietary fat content (g) was higher during ER than AR10 and AR20 (p<0.05). Further, the dietary protein (in %) was significantly lower during FR compared to BR (p<0.01), ER (p<0.05), AR10 (p<0.05) and AR20 (p<0.05).

**Conclusion:**

Ramadan had no-adverse effects on the 5-jump performance, alertness, or mood states in physically active young men. However, the sleep duration was shorter and the sleep quality was improved following compared to during Ramadan. The fractional intake of fat also increased at the expense of carbohydrate during Ramadan, and the protein intake was lower at the beginning of Ramadan than before, at the end of and after Ramadan.

## Introduction

Ramadan is a month during which healthy adult and pubescent Muslims must abstain from all types of liquid and nutrient intake from sunrise to sunset. Based on the detection of the crescent moon, the length of the month is either 29 or 30 days. Each year, Ramadan progresses forward 11 days, according to the Gregorian calendar, and it can therefore occur in either summer or winter seasons. Every day before dawn, observers take a pre-fast meal called «*Sahour*» and then begin fasting until the evening meal of "*Iftar*". Food and drink can be consumed between the *Iftar* and the *Sahour* meals [1,2].

During recent years, Muslim athletes have faced international competitions during (*e*.*g*., the Olympic Games of 2012 and the World Soccer Cup of 2014) or just after (*e*.*g*., the summer Olympic Games of 2016, the Mediterranean Games of 2018, and the World Soccer Cup of 2018) Ramadan. Thus, Muslim athletes may have to compete or to prepare for important competitions while fasting during Ramadan. Farooq et al. [3] commented that most Muslim footballers participating to the 2012 Olympic Games in London believed that Ramadan fasting would negatively affect their performance, and plainly to optimize the achievements of observant competitors, sports scientists, coaches and athletes need detailed knowledge of the evolution of performance and human behavior during and after Ramadan.

There have already been considerable researches on the effects of Ramadan observance on physical performance and behavior [4]. Specific impairments of physical performance were evident during the Wingate test, repeated sprint exercises, 40-m sprint and agility tests [5–7]. However, other studies have reported no significant changes in short-term maximal performance (*e*.*g*., in the squat jump and countermovement jump, and in sprinting) during the fasting period [8–10]. Such contradictions between results probably reflect, among other factors, differing individual impacts of Ramadan observance upon sleep, diet and training patterns, and the extent of adjustments in lifestyle made by the individual or advised by coaching staff. Changes in sleep and diet [11–13] seem key factors influencing training, competitive performance and recovery [14,15].

Several previous studies reported that Ramadan observance caused disturbances in both the quantity and the quality of sleep [6,13,16,17] as well as modifying daily food and water intake [12,18]. Recently, Aziz et al. [19] further suggested that Ramadan fasting can affect short-term maximal performance even if the dietary intake, hours of sleeping and training loads are well maintained. Another possible adverse influence comes from changes in alertness and/or mood states. Roky et al. [16] suggested that a perturbation of mood states and mental activity during Ramadan could contribute to declines in performance. Others, also, have reported increased feelings of malaise, lethargy, fatigue and mood swings which could compromise the athlete's ability to sustain high-intensity exercise [5,20,21].

Chaouachi et al. [8] found no significant differences in squat jump, countermovement jump, 30-s repeated jumps and total fatigue score before, during and after Ramadan. Abedelmalek et al. [22] observed no significant changes of muscle power during the Wingate test, and Boukhris et al. [23] recently reported no significant effect of Ramadan observance on short-term maximal performance during the 5-m shuttle run test. However, two weeks after Ramadan, Zerguini et al. [6] noted that performance of the agility and the 20-m sprint tests were below the values recorded BR. In contrast, Kirkendall et al. [10] reported an improvement of vertical jump and agility test scores after compared to BR. Chamari et al. [24] also showed an increase in the accuracy of trained cyclists as tested by rapid visual information processing two weeks after Ramadan compared to BR.

In view of these conflicting results, the present study examined the evolution of short-term maximal performance, alertness, dietary intake, sleep pattern and mood states during Ramadan observance, comparing values obtained before and during Ramadan with those found ten (AR10) and twenty (AR20) days after Ramadan, in physically active men who were not constrained by the communal living, heavy training schedules and coach-imposed lifestyles of elite athletes. We hypothesized that Ramadan observance might cause changes in sleep quantity and/or quality and dietary composition without having a significant impact on 5-jump test performance, alertness or mood state.

## Materials and methods

### Participants and experimental setting

The length of each fasting day was approximately 16 hours and the study was conducted during the 2016 Ramadan month. The average temperature and humidity were around 28°C and 50% BR, 31–32°C and 49% during Ramadan and 31°C and 47% after Ramadan. Participants were twelve physically active men (age: 21.9±2.4years, height: 1.76±0.06m, body-mass: 72.6±7.8 kg), who voluntarily participated in the study. None suffered from any injury or illness, all were non-smokers, and none were taking any medication. All were living at home and they had received no specific advice on methods of minimizing the effects of Ramadan observance. They were not members of specific sports teams, but practiced a moderate amount of regular physical activity (typically fast walking and jogging for at least 3 hours per week). Their normal time for exercising was 17h00, and this was not modified either in timing or in volume during Ramadan. None of the group normally napped during the daytime, and no naps were introduced into the daily schedule during Ramadan.

The study was conducted according to the Code of Ethics for Human Experimentation of the World Medical Association and the Declaration of Helsinki [25]. Participants were free to withdraw from the study at any time without further consequences. Participants were informed in detail about the design of the study, including the potential risks and benefits of included procedures, before providing their informed written consent to participate. The Ethics Committee of the High Institute of Nursing of University of Sfax, Tunisia, has approved the protocol before the beginning of the assessments.

### Experimental design

After a familiarization session, participants performed tests on five occasions: 15-days BR, during the first (FR) and last 10 days of Ramadan (ER), and 10 and 20 days after Ramadan (AR10 and AR20). Each test session was conducted at 17h00, beginning with the digit-cancellation test, and followed by the 5-jump test after a 5-min warm-up. During each test visit, participants also completed the Pittsburgh Sleep Quality Index (PSQI) [26] and the Profile of Mood States (POMS) [27]. Further, the amount and type of food and fluids consumed by each participant was recorded.

### 5-jump test

The 5-jump test evaluates the explosive power of the lower limbs [28]. Participants make five consecutive strides, with the feet together at the start and the end of the test. Performance is measured with a tape-measure, from the front edge of the participant’s feet at the starting position to the rear edge of the feet at the final position. Three repetitions are performed, with a recovery interval of 2 min, and the best result is retained to calculate the average stride length according to the formula:

Averagestride(m)=Totaldistance(m)/5

### Dietary intake

Participants noted all meals consumed throughout the experimental period, noting recording both the amounts and types of food and fluid consumed, and they were also interviewed by a nutritionist. Findings were analyzed using the software program Bilnut (Nutrisoft Bilnut: Food Survey Program version 2.01).

### The Pittsburgh Sleep Quality Index (PSQI)

A validated Arabic version of the PSQI was used to assess subjective sleep quality [26]. Nineteen questions examined the sleep quality, sleep duration, sleep latency, sleep disturbances, sleep efficiency, daytime dysfunction, and the use of sleep medications. The total score for the PSQI ranges from “0” to “21”, with “0” indicating no difficulty and “21” indicating severe difficulty in all areas of sleeping.

### Profile of Mood States (POMS)

The French version of the self-report POMS questionnaire was used to evaluate the subjective mood states [27]. This questionnaire assesses seven mood states (*i*.*e*., tension, depression, anger, vigor, fatigue, confusion and interpersonal relationships) from 65 adjectives. Responses to each item range from “0” (not at all) to “4” (extremely), with higher scores thus indicating a more negative mood state.

### The digit-cancellation test

The digit-cancellation test provides a highly practical and user-friendly simple assessment of various aspects of prefrontal cortex function, including information processing speed, attentional focus and executive functioning [29].

Participants performed the test over one minute by deleting target numbers (*i*.*e*., numbers composed by three grouped digits) on a sheet of randomly arranged possibilities. The sum of the correctly deleted numbers is tallied.

### Statistical analyses

Statistical analyses were performed using SPSS version 21.0 software (SPSS Inc., Chicago, Illinois, USA). Using the software G*power [30] and procedures suggested by Beck [31], the minimum sample size was *a priori* calculated. α and p values were set at 0.05 and 0.95 respectively. Effect sizes were estimated as 0.46 after discussions between the authors and based on the study of Herrera [13]. In total, to reach the desired power, data were required from at least 11 participants.

Means, SDs (standard deviations) are reported in Table 1 and SEs (Standard errors), reported in Tables 2, 3 and 4 were calculated for each variable. When the Shapiro-Wilk *W*-test revealed that data were normally distributed, parametric tests were performed. Data for the 5-jump test, PSQI, energy intake, carbohydrate (g and %), fat (% and g), protein (g) and the digit-cancellation test were analyzed using a one-way repeated measures ANOVA [covering the 5 test periods]. When appropriate, significant differences between means were tested using the Bonferroni post-hoc test. Effect sizes were calculated as partial eta-squared *ɳ*_*p*_^*2*^, with values of ηp^2^ ≥ 0.01 indicated small, ≥0.06 medium and ≥0.14 large effects respectively [32]. When data was not normally distributed (for the POMS questionnaire and dietary protein %), a Friedman non-parametric analysis of variance (ANOVA) was used. When significant, pair-wise comparisons were made using a Wilcoxon test; p values were adjusted using the Bonferroni method, and the effect size was estimated by Kendall’s coefficient of concordance. Significant differences were accepted for all analyses at the level of p<0.05.

**Table 1:** Daily dietary intake (mean±SD) recorded before (BR), at the beginning (FR), at the end (ER) and 10-days (AR10) and 20-days (AR20) after Ramadan.

	BR	FR	ER	AR10	AR20
**Energy intake (kJ/day)**	10929±2008	11016±1082	9987±1908	9343±2745	9883±2201
**Carbohydrate**					
**(g/day)**	329±49	313±77	275±68	295±89	312±73
**(%)**	51±7	47±7	46±8	53±3*	52±4*
**Protein**					
**(g/day)**	80±22	76±11	75±20	68±19	73±14
**(%)**	12±2	11±1^&^	12±2^+^	12±2^+^	12±2^+^
**Fat**					
**(g/day)**	107±34	120±18^#^	112±28	85±29	92±26
**(%)**	36±7	41±6	40±9	35±3	35±4

*: Significant difference compared to ER.

#: significant higher compared to AR10.

&: Significant difference compared to BR.

+: Significant difference compared to FR.

**Table 2:** Subjective sleep quality (mean±SE) as estimated by the Pittsburgh Sleep Quality Index (PSQI). Values before (BR), at the beginning (FR), at the end (ER) and after 10-days (AR10) and 20-days of Ramadan (AR20).

	BR	FR	ER	10 AR	20 AR
**Subjective sleep quality (AU)**	1.07±0.25	1.27±0.21&	1.13±0.22+	2.00±0.20&+*	1.67±0.21&+*#
**Sleep latency (min)**	15.42±0.74	13.75±1.25	13.75±1.39	16.42±2.22	16.17±1.04
**Sleep duration (h)**	7.33±1.30	7.83±1.40&	7.50±1.73+	6.50±1.62&+*	6.67±1.78&+*
**Habitual sleep efficiency (%)**	95.37±2.03	93.42±3.49	95.77±2.54	95.37±2.03	96.76±1.74
**Sleep disturbances (AU)**	1.08±0.13	0.58±0.13&	0.58±0.13&	0.75±0.12&+*	0.75±0.12&+*
**Use of sleeping medication (AU)**	0.08±0.07	0.00±0.00	0.00±0.00	0.08±0.07	0.08±0.07
**Daytime dysfunction (AU)**	0.75±0.22	0.58±0.23	0.33±0.17	0.75±0.22	0.33±0.13
**Global PSQI Score (AU)**	4.92±0.45	4.12±0.48	4.00±0.64	6.58±0.66	5.58±0.50

&: Significant difference compared to BR.

+: Significant difference compared to FR.

*: Significant difference compared to ER.

#: Significant difference compared to AR10.

AU: arbitrary units.

**Table 3:** Mood states parameters (mean±SE) as estimated by the Profile of Mood States. Values before (BR), at the beginning (FR), at the end (ER) and after 10-days (AR10) and 20-days of Ramadan (AR20).

	BR	FR	ER	AR10	AR20
**Tension (AU)**	9.67±1.87	8.83±1.81	10.17±1.89	8.92±1.45	10.17±1.37
**Anger (AU)**	11.92±2.96	8.42±2.63	9.92±3.01	10.17±1.86	10.5±2.18
**Confusion (AU)**	7.58±1.2	7.17±1.36	7.25±1.19	8.42±1.01	7.75±1.15
**Depression (AU)**	9.08±2.72	8.67±3.34	7.58±3.13	10.08±2.52	10.08±2.72
**Fatigue (AU)**	7.42±1.73	6.17±1.71	6.67±1.81	7.17±1.31	7±1.62
**Interpersonal relationships (AU)**	17.5±1.86	15.83±1.98	15.67±1.90	14.92±1.80	14.42±1.92
**Vigor (AU)**	19.25±1.85	16.58±2.22	16.17±2.04	16.25±1.85	16±1.72
**Total score (AU)**	26.42±9.84	22.67±10.48	25.42±10.28	28.5±6.74	29.5±8.10

AU: arbitrary units.

**Table 4:** Average stride recorded during the 5-jump test and number of the correct responses registered during the digit cancellation test (mean±SE) before (BR), at the beginning (FR), at the end (ER) and 10-days (AR10) and 20-days (AR20) after Ramadan.

	BR	FR	ER	AR10	AR20
**Average stride length (m)**	2.66±0.05	2.66±0.06	2.68±0.04	2.68±0.05	2.68±0.04
**Correct responses**	66.25±2.96	62.67±4.17	63.17±3.38	63.92±2.78	65.17±2.93

## Results

### Dietary intake

The total daily energy intake, the dietary carbohydrate (in g), the dietary protein (in g) and the fractional contribution of fat (in %) remained the same during all periods of observation (p>0.05). However, the fractional contribution of carbohydrate (in %) was lower during ER than in AR10 and AR20 (p<0.05), and the dietary fat intake (in g) was significantly higher during FR than AR10 (p<0.05), see Table 1. Further, the fractional contribution of protein (in %) was significantly lower during FR compared to BR (p<0.01), ER (p<0.05), AR10 (p<0.05) and AR20 (p<0.05).

### The Pittsburgh Sleep Quality Index (PSQI)

The PSQI results are presented in Table 2. There was a significant main effect of time periods for the subjective sleep quality (Test = 17.02; p<0.01; Kendall’s W = 0.3), with lower values BR compared to FR (p<0.05), AR10 (p<0.01) and AR20 (p<0.01). Also, the subjective sleep quality recorded at FR was greater than at ER (p<0.05) and lower than at AR10 (p<0.01) and AR20 (p<0.05). The subjective sleep quality recorded at ER was significantly poorer than at AR10 (p<0.001) and AR20 (p<0.01), and the sleep quality recorded at AR10 was significantly greater than at AR20 (p<0.05).

The Friedman test revealed a significant main effect of time periods for sleep duration (Test = 15.67; p<0.01; Kendall’s W = 0.33). Compared to BR, the pair-wise comparison indicated that the sleep duration (*i*) increased at FR (p<0.05) and (*ii*) decreased at AR10 (p<0.01) and AR20 (p<0.05). Also, the sleep duration was significantly shorter (*i*) at ER (p<0.05), AR10 (p<0.01) and AR20 (p<0.01) than at FR, and (*ii*) at AR10 (p<0.01) and AR20 (p<0.05) compared to ER.

There was, also a significant main effect of time periods for sleep disturbances (Test = 13.38; p<0.01; Kendall’s W = 0.28). Sleep disturbances were significantly greater (*i*) at BR compared to FR (p<0.01), ER (p<0.01), AR10 (p<0.05) and AR20 (p<0.05) and (*ii*) at AR10 and AR20 compared to FR and ER (p<0.05).

Further, there was a significant main effect of time periods for the total PSQI score (F = 6.23; p<0.001; *ɳ*_*p*_^*2*^ = 0.36), with higher total scores at AR10 compared to FR (p<0.01) and ER (p<0.001).

However, no-significant main effect was seen for sleep latency (Test = 3.82; p>0.05; Kendall’s W = 0.08), habitual sleep efficiency (Test = 1.18; p>0.05; Kendall’s W = 0.02), use of sleeping medication (Test = 2.4; p>0.05; Kendall’s W = 0.05) or daytime dysfunction (Test = 8.12; p>0.05; Kendall’s W = 0.17).

### Profile of Mood States (POMS)

The statistical analysis revealed no-significant main effects of time period for tension (Test = 1.41; p>0.05; Kendall’s W = 0.03), anger (Test = 3.02; p>0.05; Kendall’s W = 0.06), confusion (Test = 1.41; p>0.05; Kendall’s W = 0.03), depression (Test = 5.06; p>0.05; Kendall’s W = 0.11), fatigue (Test = 4.07; p>0.05; Kendall’s W = 0.08), vigor (F = 1.07; p>0.05; *ɳ*_*p*_^*2*^ = 0.09), or interpersonal relationships (F = 1.63; p>0.05; *ɳ*_*p*_^*2*^ = 0.12), nor for the total POMS score (Test = 2.66; p>0.05; Kendall’s W = 0.06), see Table 3.

### The digit cancellation test

There was no-significant main effect of time period (F = 1.15; p>0.05; *ɳ*_*p*_^*2*^ = 0.09) for the number of correct responses during the digit-cancellation test, see Table 4.

### 5-jump test

There was no-significant main effect of time period (F = 0.37; p>0.05; *ɳ*_*p*_^*2*^ = 0.03) for the average stride length during the 5-jump test, see Table 4.

## Discussion

The main conclusion from the present study is that in a group of young men performing the moderate volume of regular physical activity commonly recommended for maintenance of good health (>3 h/week) [33], the average stride length during the 5-jump test, alertness and self-reported mood state did not change during and following the intermittent fasting of Ramadan observance.

In agreement with the present results, Bouhlel et al. [34], Zerguini et al. [6] and Chaouachi et al. [8] also found that Ramadan observance did not affect performance during the vertical jump test. Likewise, Baklouti et al. [35] reported that performance of the 5-jump test was unaffected by Ramadan observance in soccer players who maintained their training during Ramadan.

The unchanged performance of the 5-jump test could be explained in part by the short duration of this test. Performance decrements have been seen during longer periods of exercise that induce a sensation of fatigue [5,19,36].

In agreement with Roky et al. [21] and Sweileh et al. [37], the present data also show alertness (as assessed by correct responses in the digit cancellation test) was unchanged during and after Ramadan. Previous studies saw no changes in alertness, mood states or physical performance [5,21,37]. The present study also found unchanged mood states during and after Ramadan observance. It has been suggested that changes in mood and mental alertness could adversely affect physical performance during Ramadan [5,21,37], and the unchanged 5-jump performance could be attributed, in part, to the maintenance of mood states and alertness.

The present results showed that the total daily energy intake, the dietary carbohydrate content, the dietary protein content and the fractional contribution of fat were similar between BR, FR, ER, AR10 and AR20. However, changes were seen for the fractional contribution of carbohydrate (*i*.*e*., lower at ER *vs*. AR10 and AR20), the dietary fat intake (*i*.*e*., higher at FR *vs*. AR10) and the fractional contribution of protein (*i*.*e*., lower at FR *vs*. BR, ER, AR10 and AR20). In agreement with these results, a previous study reported that the total energy intake [22] was unchanged during as compared to BR. Likewise, in soccer players, Chtourou et al. [36] and Aloui et al. [38,39] showed that the total daily energy intake and the fractional contribution of fat were maintained during Ramadan as compared to BR, and Hammouda et al. [40] reported that the carbohydrate content and the dietary protein content were maintained during Ramadan compared to BR. Abdelmalek et al. [22] and Aloui et al. [38,39] suggested that a stable total energy intake between BR and during Ramadan could help to maintain physical performances over Ramadan. In contrast, Aziz et al. [19] argued that inadequacies of food and fluid intake could contribute to poor performance during Ramadan.

The present PSQI results showed a number of impacts of Ramadan observance upon sleep. The subjective sleep quality was lower (*i*) at BR compared to FR, AR10 and AR20, (*ii*) at ER compared to FR, (*iii*) at AR10 and AR20 compared to FR and (*iv*) at AR20 compared to AR10. The sleep duration was (*i*) reduced at AR10 and AR20 and enhanced at FR in comparison to BR, (*ii*) reduced at ER, AR10 and AR20 compared to FR and (*iii*) reduced at AR10 and AR20 compared to ER. Sleep disturbances were significantly greater (*i*) at BR compared to FR, ER, AR10 and AR20 and (*ii*) at AR10 and AR20 compared to FR and ER. The total score of the PSQI was also higher at AR10 compared to FR and ER. However, Ramadan had no significant effect on sleep latency, habitual sleep efficiency, use of sleeping medications or daytime dysfunction.

In agreement with the present study, Boukhris et al. [23] reported no significant changes of sleep latency, habitual sleep efficiency, use of sleeping medication or daytime dysfunction between BR, FR, ER, AR10 and AR20. Moreover, in agreement with the results of the present study, Boukhris et al. [23] reported that sleep duration was reduced AR10 and AR20 in comparison with FR and ER. In contrast, Herrera et al. [13] saw no significant changes of sleep quality during Ramadan.

Taken together the results of the present study suggest that although sleep duration was shorter, its quality was better after Ramadan compared to during and BR. Sleep is important both for optimal physical performance and for speeding recovery processes [41,42]. Participants in the present study showed a significant decrease in sleep disturbances during Ramadan compared to BR. As suggested by Boukhris et al. [23], the maintenance of sleep quality and efficiency and the reduction of sleep disturbances could, in part, contribute to the unchanged short-term maximal performance, alertness and mood states observed during Ramadan.

Using objective rather than subjective measurements, but in agreement with the results of the present study, Chamari et al. [24] found that the mean sleep duration of trained cyclists decreased from around 7 hours at BR and ER to less than 6 hours two weeks after Ramadan. However, the duration of deep and Rapid Eye Movement sleep stages progressively decreased during their study, to reach significantly lower values than BR two weeks after Ramadan. The authors explained their observations by an increase of training load after Ramadan and/or delayed effects of Ramadan. Likewise, Zerguini et al. [6] saw significant changes in the sleep duration two weeks after Ramadan in comparison to before and during Ramadan, but they did not show a significant alteration of sleep quality after Ramadan.

### Limitations

The study presents some limitations: (*i*) reliance on subjective rather than objective methods for the measurement of sleep during all experimental periods and (*ii*) the absence of a control group that did not observe Ramadan. The lack of non-fasting controls is typical of most studies of Ramadan, and reflects ethical issues in Muslim majority countries such as Tunisia. In such environments, it is unfortunately not possible to recruit non-fasting study participants. Further, body mass was only measured before Ramadan; if there was a substantial decrease of body mass during or following Ramadan (this is not usually the case), it could have affected the performance measures involving the displacement of body mass. Other issues are the measurement of body-composition, the determination of the glycemic index of foods, the types of fat consumed (*i*.*e*., saturated or unsaturated fat, animal or vegetal fat), and any changes in the type of food consumed, all of which offer opportunities for further and more broadly ranging studies. Finally, the findings only apply to young men engaging in a moderate volume of weekly physical activity, without the stresses inherent in competition, heavy training and the need to follow the dictates of coaches.

## Conclusions

In conclusion, Ramadan fasting did not affect the 5-jump performance, alertness or mood state of young men performing the moderate volumes of physical activity suggested in public health recommendations. However, their sleep duration was shortened and the sleep quality improved following compared to during Ramadan. The fractional intake of fat also increased at the expense of carbohydrate during Ramadan, and the protein intake was lower at the beginning of Ramadan compared to BR, at ER and following Ramadan. Active individuals who are observing Ramadan are well advised to maintain their normal diet and sleep patterns as far as possible in order to avoid any deteriorations of physical performance or behavior during and after Ramadan observance, but with such precautions, it seems that disturbances of function can be minimal.

## Supporting information

S1 Dataset (XLSX)
